# Three-dose vaccination elicits neutralising antibodies against SARS-CoV-2 Omicron variant

**DOI:** 10.1016/S0140-6736(22)00092-7

**Published:** 2022-01-19

**Authors:** Mary Wu, Emma C Wall, Edward J Carr, Ruth Harvey, Hermaleigh Townsley, Harriet V Mears, Lorin Adams, Svend Kjaer, Gavin Kelly, Scott Warchal, Chelsea Sawyer, Caitlin Kavanagh, Christophe J Queval, Yenting Ngai, Emine Hatipoglu, Karen Ambrose, Steve Hindmarsh, Rupert Beale, Steve Gamblin, Michael Howell, George Kassiotis, Vincenzo Libri, Bryan Williams, Sonia Gandhi, Charles Swanton, David LV Bauer

**Affiliations:** 1 The Francis Crick Institute, 1 Midland Road, London, UK; 2 National Institute for Health Research (NIHR) University College London Hospitals (UCLH) Biomedical Research Centre and NIHR UCLH Clinical Research Facility, London, UK; 3 Worldwide Influenza Centre, The Francis Crick Institute, 1 Midland Road, London, UK; 4 University College London, Gower Street, London; 5 Department of Infectious Disease, St Mary’s Hospital, Imperial College London, London; 6 Genotype-to-Phenotype UK National Virology Consortium (G2P-UK)

The SARS-CoV-2 B.1.1.529 (Omicron) Variant of Concern (VOC) was first detected in Southern Africa in November 2021 and its BA.1 sub-lineage is now dominant in the United Kingdom. Omicron BA.1 contains 32 coding changes in its Spike protein (Figure 1A; appendix p 2) and it is unclear to what extent its spread is driven by an intrinsic increase in transmissibility or escape from prior infection- and vaccine-induced immunity.

In the United Kingdom, the BNT162b2 (Pfizer-BioNTech) and AZD1222 (ChAdOx1 nCoV-19, Oxford-AstraZeneca) vaccines were administered as part of a primary two-dose course and a subsequent third dose of either BNT162b2 (Pfizer-BioNTech) or mRNA1273 (Moderna) vaccine has been administered since September 2021. To determine the ability of vaccine-induced antibodies to neutralise the Omicron variant and compare this to our previous measurements of VOC neutralisation by BNT162b2 (Wall, Wu et al., Lancet 2021a) and AZD1222 (Wall, Wu, Harvey et al., Lancet 2021b), we carried out a third analysis of the Legacy study cohort (NCT04750356). The Legacy study was established in January 2021 by University College London Hospitals and the Francis Crick Institute to track serological responses to vaccination during the national COVID-19 vaccination programme in healthy staff volunteers recruited prospectively, or following a positive COVID test, after vaccination. A description of the methods and clinical cohort are available in the appendix. The Legacy study was approved by London Camden and Kings Cross Health Research Authority Research and Ethics committee (IRAS number 286469) and is sponsored by University College London Hospitals.

Using a high-throughput live SARS-CoV-2 neutralisation assay, we determined NAb titres (NAbTs) for 620 serum samples from 364 unique participants (Table 1 and Figure 2; appendix p 3) against the Omicron VOC, as well as the Alpha and Delta VOCs, for which there is significant vaccine efficacy data correlated with NAbTs (Cromer et al., Lancet Microbe, 2021).

In participants sampled 2-6 weeks following two-dose vaccination with BNT162b2, the majority (166/199, 83%) had a quantifiable NAbT against Omicron (median IC_50_ = 122, [IQR 46-173]), which was 7-fold [95%CI: 6.3-7.4] lower than NAbTs against Alpha (median IC_50_ = 600, [IQR 384-1141]) and 3-fold [95%CI: 2.8-3.3] lower than NAbTs against Delta (median IC_50_ = 301, [IQR 171-572]) (Figure 1B; appendix p 2). However, when sampled 12-16 weeks following two-dose vaccination with BNT162b2, only around half had a quantifiable NAbT against Omicron (69/136, 51%) — whereas nearly all still had a quantifiable NAbT against Alpha (131/136, 96%) and Delta (132/136, 97%). The drop in Omicron NAbT in the 10 weeks after the second dose was significant (χ^2^ p<0.0001).

The same analysis of participants following two-dose vaccination with AZD1222 found that less than half had a quantifiable NAbT against Omicron 2-6 weeks following their second dose (25/68, 37%), dropping further to 5/26 (19%) at 12-16 weeks following their second dose — whereas most had a quantifiable NAbT against Alpha (59/68, 87%) and Delta (52/68, 76%) at 2-6 weeks following their second dose of AZD1222 (Figure 1B; appendix p 2). Notably, NAbTs following AZD1222 vaccination differed significantly according to whether participants reported experiencing COVID symptoms (χ^2^ p<0.0001): those who had received two doses of AZD1222 and had not experienced COVID symptoms at any point before their second vaccine dose largely had no detectable NAb response against Omicron (29/40, 73%) (Figure 1C and Table 2; appendix p 2 and p 4). In contrast, only a minority of those who had received two doses of BNT162b2 and had not experienced prior COVID symptoms had no detectable NAb response against Omicron (24/147, 16%), though the median NAbT against Omicron of this group was significantly lower than those BNT162b2 recipients who did report prior COVID symptoms (median IC_50_ = 92 [IQR 42-158] vs. 165 [122-387], p<0.0001) — consistent with our previously-published results against the Delta variant (Wall, Wu, Harvey, et al., Lancet, 2021b).

Following two doses of vaccine, 26 participants experienced subsequent “breakthrough” SARS-CoV-2 infection (April-November 2021, 24/26, 92%, likely Delta infections, see Methods in Appendix) and presented for a study visit 1-7 weeks following a positive COVID-19 test (Figure 1D; appendix p 2). All participants, irrespective of vaccine type, were able to subsequently neutralise the Omicron variant (median IC_50_ = 573, [IQR 310-655]).

In September 2021, the United Kingdom initiated a targeted third-dose “booster” campaign for those in JCVI Priority Groups 1-9 who had received their second dose more than 6 months prior, which included healthcare workers, those over 50 years of age, or those classed as clinically vulnerable. Participants were invited for a study visit at the time of their third dose (n=80, median days since second dose = 192 [IQR 188-202]), and following their third dose (n=85, median days since third dose = 20 [IQR 18-22]; median age = 53 years [IQR 45-59]). All participants received BNT162b2 for all three doses (Figure 1E; appendix p 2). Prior to receiving their third dose, a minority of participants had a detectable NAbT against Omicron (34/80, 42%), whereas following their third dose nearly all participants neutralised Omicron (82/85, 96%) with a median IC_50_ of 332 [IQR 193-596]. Following a third dose of BNT162b2, NAbTs against Omicron at ~3 weeks post-vaccination were only 4-fold lower (95%CI 3.3-4.5) than against Alpha and only 2-fold lower (95%CI 1.7-2.0) than against Delta.

Finally, we considered whether two synthetic monoclonal antibody treatments available in the United Kingdom were able to neutralise the Omicron VOC: Xevudy (sotrovimab, Vir/GSK) was able to neutralise Omicron (geometric mean IC_50_ = 385 ng/ml [95%CI: 354-419]), whereas Ronapreve (casirivimab/imdevimab, Regeneron) did not — even at concentrations up to 300,000 ng/ml (Figure 1F; appendix p 2). While sotrovimab was 6- to 8-fold less effective at neutralising Omicron than Delta or Alpha, the mean serum concentration of sotrovimab 29 days following a 500 mg infusion (24.5 μg/mL, Summary of Product Characteristics for Xevudy, UK MHRA) is 64-fold higher than the *in vitro* IC_50_ measured here.

In summary, our results show that two vaccine doses — and of AZD1222 in particular — are insufficient to generate a strong NAbT against the SARS-CoV-2 Omicron VOC. Participants who experienced a COVID-19 infection before or after 2-dose vaccination generated higher NAbTs than those who did not experience a COVID-19 infection — as did those who received a third dose of BNT162b2, who produced consistently high NAbTs against Omicron (and Alpha and Delta). These findings have two important implications. Firstly, they suggest that current vaccines, encoding the ancestral Spike protein first detected in Wuhan, China, may still induce an equivalent NAbT against Omicron than infection with other recent VOCs, supported by considerations of the antigenic distance between ancestral and VOC spikes (Dejnirattisai et al., Lancet, 2021; Cele et al., Nature, 2021). Secondly, whereas each Spike variant appears to induce the highest NAbT to itself with defined hierarchy of cross-reactivity (Faulkner et al., eLife, 2021; Reynolds et al., Science, 2021), we observe that the differential in the cross-recognition of heterologous Spikes is substantially reduced following booster vaccination, in line with recent reports (Dejnirattisai et al., Lancet, 2021). It will be important to dissect the features that drive this broad response across varied cohorts (vaccine type, prior infection, age, comorbidities) as future booster vaccination strategies are considered.

We also found that the monoclonal antibody sotrovimab (but not casirivimab/imdevimab) neutralised Omicron *in vitro* — albeit reduced relative to Alpha and Delta. This finding is in line with other preliminary reports (Planas et al., Nature, 2021), and is supportive of plans to prioritise the use of sotrovimab in clinically vulnerable adults following MHRA approval in December 2021. While sotrovimab likely retains activity against Omicron, our results suggest it would be prudent to evaluate any changes in its efficacy across multiple settings and dosing regimens, in support of the recent decision to study sotrovimab in the RECOVERY and PANORAMIC trials in 2022. In the meantime, however, our results also suggest that given the lack of any other available monoclonal antibody treatment for those with Omicron infections, it would be prudent to urgently consider extending the use of sotrovimab beyond those not requiring supplemental oxygen — i.e. for those who are more severely ill and would have been given casirivimab/imdevimab previously, but who are not presently approved to receive sotrovimab.

Overall, our results suggest that NAbTs against Omicron following a third vaccine dose of BNT162b2 are on a similar order to NAbTs against Delta following a second dose of BNT162b2. While changes in intrinsic viral characteristics such as tissue tropism or transmissibility might alter the precise level of NAbs that correlates with protection from illness, NAbTs measured in the laboratory remain strongest correlate of protection against symptomatic and severe illness across multiple variants (Khoury et al., Nature Medicine, 2021; Cromer et al., Lancet Microbe, 2021). Indeed, the NAbTs we observe are broadly consistent with preliminary epidemiological data of vaccine efficacy against symptomatic disease (UKHSA Variant Technical Briefing 33, Figure 10).

It is worth highlighting that comparisons between cohorts presented here are confounded by age: participants who were sampled following two doses of AZD1222 are significantly younger than participants who were sampled following two doses of BNT162b2 (median age of 35 vs 45 years, p<0.0001, see Table 1), whereas the converse is true for participants who were sampled following three doses of BNT162b2 (median age of 55 years). Comparison of this latter group to an older subset of the two-dose BNT162b2 cohort (n=88, age ≥45 years, Table 3; appendix p 5), however, shows similar results (Figure 3; appendix p 5). Should the correlation between younger age and higher NAbTs we previously observed following two dose vaccination (Wall et al., Lancet, 2021a) continue to hold true, our results would imply that absolute NAbTs following three dose BNT162b2 vaccination measured here are an underestimate of those in younger cohorts. Ultimately, our observational study is constrained by the rollout of the national COVID-19 vaccination programme: further assessment of NAbTs in younger individuals, waning of NAbTs over time, as well as the effect of third-dose vaccination in participants who previously received two doses of AZD1222 (which has formed the backbone of the global vaccination programme) will be necessary.

To conclude, the results from our cohort of healthy, working-age adults support a three-dose vaccination strategy against COVID-19 for the general population — and the broad neutralising response observed suggests urgent global action to deliver three-dose vaccination may increase population immunity against current VOCs (including Omicron), and may help prevent the emergence of new variants. Studies from diverse populations, including the elderly and clinically extremely vulnerable — such as those on haemodialysis (**Carr et al., accompanying manuscript**) or undergoing treatment for cancer (**Fendler et al., accompanying manuscript**) remain critical to understand immunity in groups that are most at risk and require a larger share of healthcare resources should they fall ill. Overall, it remains critical to monitor NAbTs over time in diverse cohorts: while many aspects of cellular immunity are at play, both reports of mortality reduction in antibody-negative adults infected with the Alpha VOC and treated with casirivimab/imdevimab (RECOVERY, Horby, et al., medRxiv 2021), and recent reports of concomitant NAb waning and increasing risk of hospitalisation or death (Katikireddi et al., Lancet, 2021) across multiple populations, suggest ongoing assessment of NAb against SARS-CoV-2 variants will continue to be part of an effective strategy against COVID-19 as the pandemic continues to evolve.

## Supplementary Material

Figure 1

## References

[R1] Cele S, Jackson L, Khoury DS (2021). Omicron extensively but incompletely escapes Pfizer BNT162b2 neutralization. Nature.

[R2] Cromer D, Steain M, Reynaldi A (2021). Neutralising antibody titres as predictors of protection against SARS-CoV-2 variants and the impact of boosting: a meta-analysis. Lancet Microbe.

[R3] Dejnirattisai W, Shaw RH, Supasa P (2021). Reduced neutralisation of SARS-CoV-2 omicron B.1.1.529 variant by post-immunisation serum. Lancet.

[R4] Faulkner N, Ng KW, Wu MY (2021). Reduced antibody cross-reactivity following infection with B.1.1.7 than with parental SARS-CoV-2 strains. Elife.

[R5] Katikireddi SV, Cerqueira-Silva T, Vasileiou E (2021). Two-dose ChAdOx1 nCoV-19 vaccine protection against COVID-19 hospital admissions and deaths over time: a retrospective, population-based cohort study in Scotland and Brazil. Lancet.

[R6] Planas D, Saunders N, Maes P (2021). Considerable escape of SARS-CoV-2 Omicron to antibody neutralization. Nature.

[R7] Horby PW, Mafham M, RECOVERY Collaborative Group (2021). Casirivimab and imdevimab in patients admitted to hospital with COVID-19 (RECOVERY): a randomised, controlled, open-label, platform trial. bioRxiv.

[R8] Reynolds CJ, Pade C, Gibbons JM (2021). Prior SARS-CoV-2 infection rescues B and T cell responses to variants after first vaccine dose. Science.

[R9] Summary of Product Characteristics for Xevudy.

[R10] UK Health Security Agency (2021). UKHSA Variant Technical Briefing 33.

[R11] Wall EC, Wu M, Harvey R (2021). Neutralising antibody activity against SARS-CoV-2 VOCs B.1.617.2 and B.1.351 by BNT162b2 vaccination. Lancet.

[R12] Wall EC, Wu M, Harvey R (2021). AZD1222-induced neutralising antibody activity against SARS-CoV-2 Delta VOC. Lancet.

