# Supplementary material for: Three-dose vaccination elicits neutralising antibodies against SARS-CoV-2 Omicron variant
Source: Lancet. Author manuscript; Available in PMC 2022 Feb 22. (PMC8769665; doi:10.1016/S0140-6736(22)00092-7)
Supplement: Figure 1 [file EMS141050-supplement-Figure_1.pdf]

Appendix for

Three-dose vaccination elicits neutralising antibodies against SARS-CoV-2 Omicron variant

Mary Wu[1]*, Emma C Wall[1,2]*, Edward J Carr[1]*, Ruth Harvey[3], Hermaleigh Townsley[1,2], Harriet V Mears[1], Lorin Adams[3], Svend Kjaer[1], Gavin Kelly[1], Scott Warchal[1], Chelsea Sawyer[1], Caitlin Kavanagh[1], Christophe J Queval[1], Yenting Ngai[4], Emine Hatipoglu[4], Karen Ambrose[1], Steve Hindmarsh[1], Rupert Beale[1,4], Steve Gamblin[1], Michael Howell[1], George Kassiotis[1,5], Vincenzo Libri[2,4] Bryan Williams [2,4], Sonia Gandhi[1,4], Charles Swanton[1,4], David LV Bauer[1,6]

Affiliations

1. The Francis Crick Institute, 1 Midland Road, London, UK
2. National Institute for Health Research (NIHR) University College London Hospitals (UCLH) Biomedical Research Centre and NIHR UCLH Clinical Research Facility, London, UK
3. Worldwide Influenza Centre, The Francis Crick Institute, 1 Midland Road, London, UK
4. University College London, Gower Street, London
5. Department of Infectious Disease, St Mary's Hospital, Imperial College London, London
6. Genotype-to-Phenotype UK National Virology Consortium (G2P-UK)

*These authors contributed equally to this work.

Senior authors: Sonia Gandhi, Charles Swanton, and David LV Bauer

Correspondence to: David LV Bauer david.bauer@crick.ac.uk

Contents

Figures 1-3

Tables 1-3

Methods

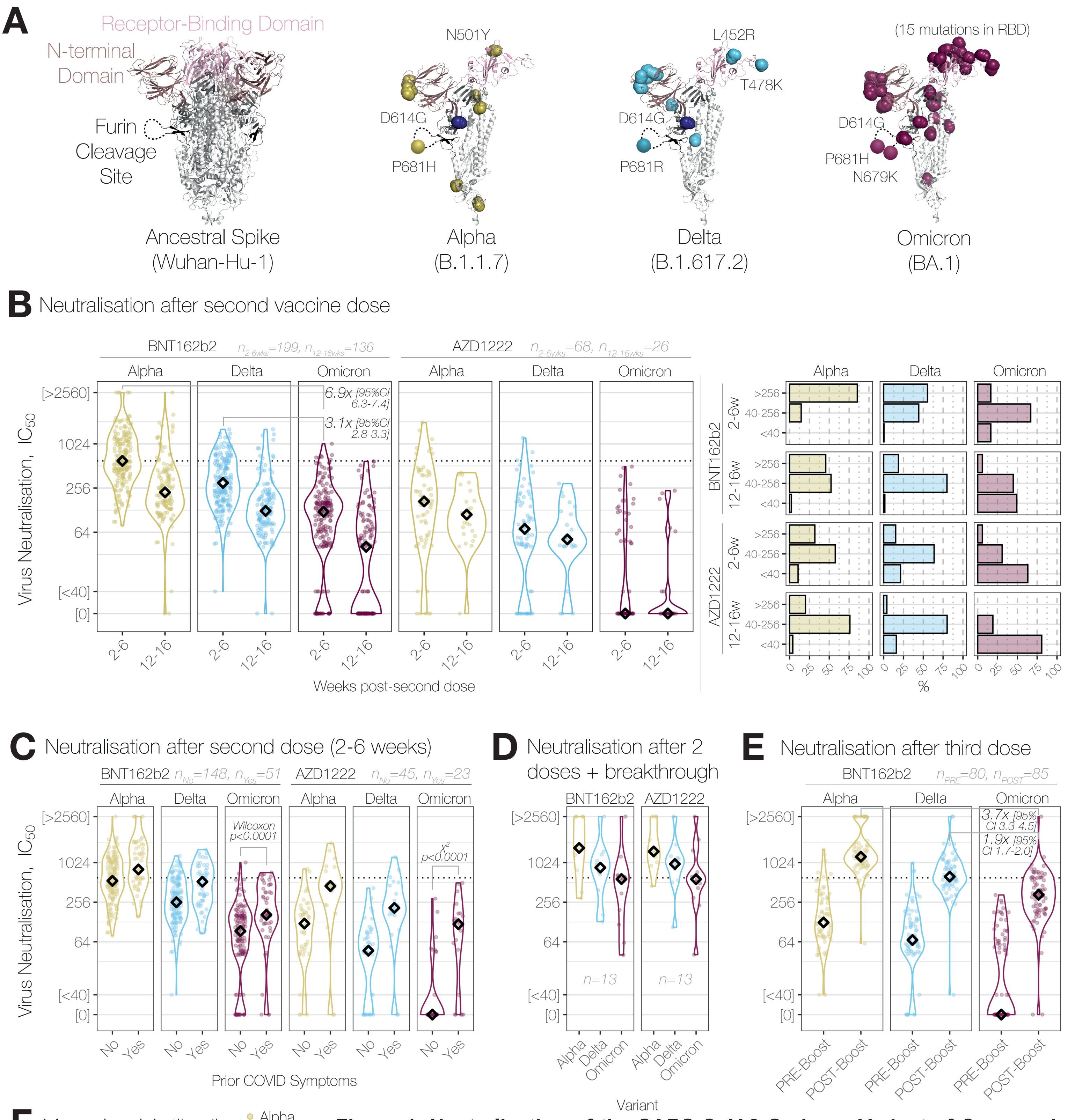

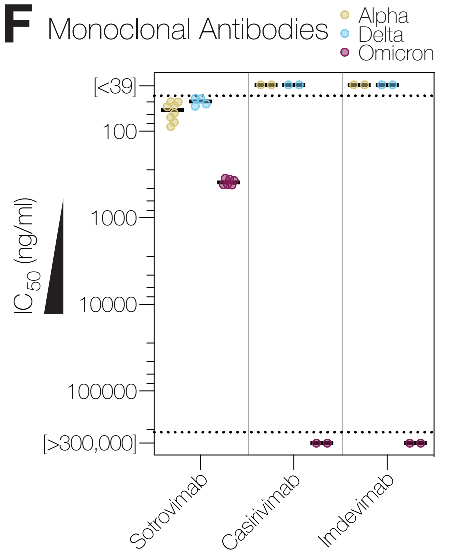

Figure 1. Neutralisation of the SARS-CoV-2 Omicron Variant of Concern in Legacy study participants. (**A**) Mutations in VOC spike proteins. (**B**) Live-virus neutralising antibody titres (NAbTs) against three SARS-CoV-2 VOCs from study participants (*n* as indicated and in **Tables 1-3**) who had received 2 doses of BNT162b2 or AZD1222 at 2-6 and 12-16 weeks following vaccination. NAbTs are expressed as serum fold-dilution required to achieve 50% virus neutralisation (IC_{50}); samples below the quantitative ([<40]) and absolute ([0]) limits of detection are plotted, and also summarised in bar plots at right after grouping into 3 response levels. (**C**) NAbTs following 2 doses of BNT162b2 or AZD1222, stratified according to participants' report of prior COVID symptoms. (**D**) NAbTs 2-6 weeks following a "breakthrough" SARS-CoV-2 infection subsequent to 2 doses of BNT162b2 or AZD1222. (**E**) NAbTs 2-6 weeks following a third dose "booster" of BNT162b2 in eligible participants who had received 2 prior doses of BNT162b2. (**F**) Neutralisation titres of monoclonal antibodies against VOCs, expressed as concentration (ng/ml) required to achieve 50% virus neutralisation (IC_{50}).

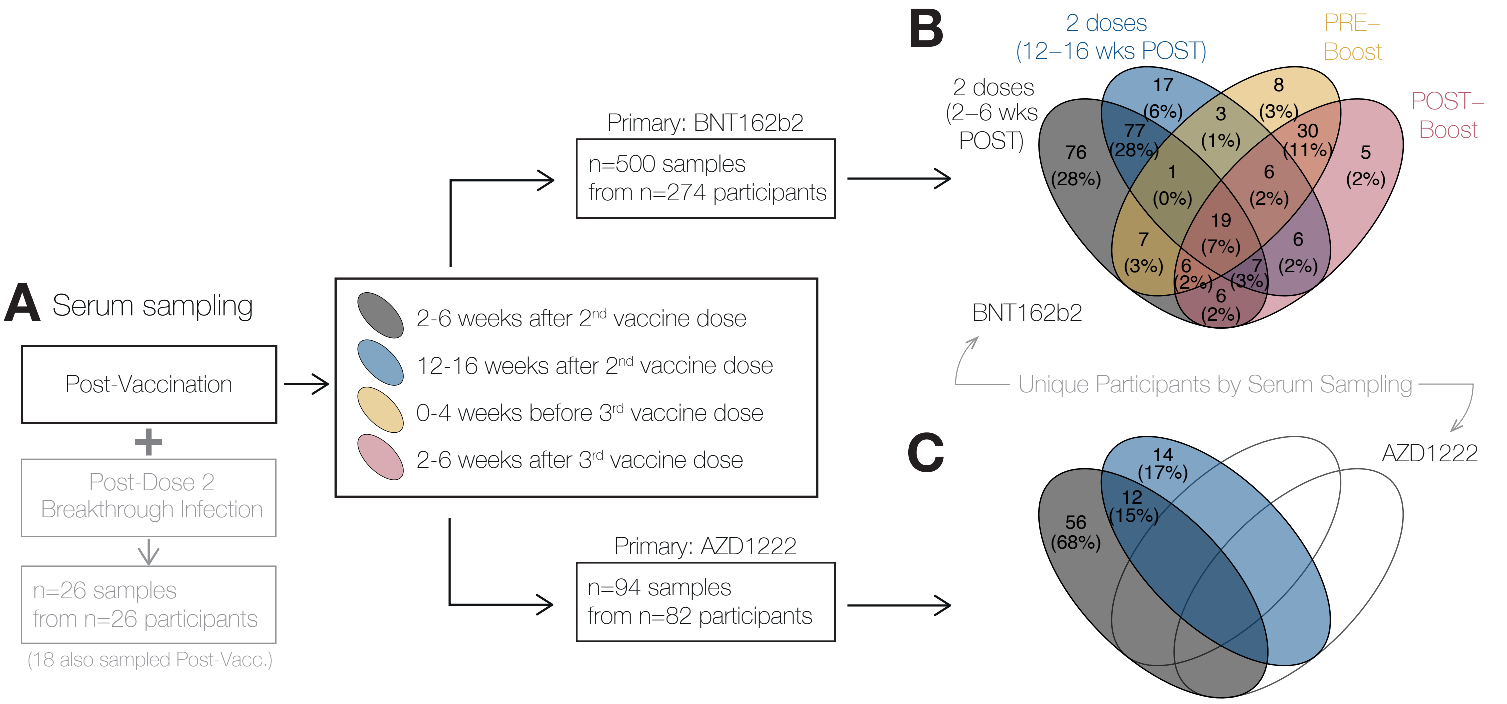

Figure 2. Diagram illustrating (**A**) serum samples collected, and number of unique participants and overlap between serum sampling, stratified by primary (**B**) BNT162b2 and (**C**) AZD1222 vaccine course.

(Refers to Figure 1B) 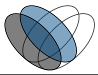 *(Refers to Figure 1D)* 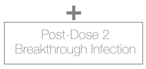

(Refers to Figure 1E) 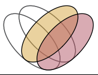

	2 Vaccine Doses				2 Vaccine Doses + Breakthrough		3 Vaccine Doses	
	BNT162b2		AZD1222		BNT162b2	AZD1222	BNT162b2	
	2-6 wks POST-	12-16 wks POST-	2-6 wks POST-	12-16 wks POST-	1-7 wks POST-	1-7 wks POST-	4 to 0 wks PRE-	2-6 wks POST-
	n = 199	n = 136	n = 68	n = 26	n = 13	n = 13	n = 80	n = 85
Site								
Crick	94 (47%)	84 (62%)	68 (100%)	26 (100%)	10 (77%)	13 (100%)	58 (72%)	54 (64%)
UCLH	105 (53%)	52 (38%)	0 (0%)	0 (0%)	3 (23%)	0 (0%)	22 (28%)	31 (36%)
Sex								
Female	136 (68%)	92 (68%)	43 (63%)	15 (58%)	6 (46%)	5 (38%)	59 (74%)	64 (75%)
Male	63 (32%)	44 (32%)	25 (37%)	11 (42%)	7 (54%)	8 (62%)	21 (26%)	21 (25%)
Median age (years) [IQR]	42 [33-52]	41 [32-52]	34 [30-42]	36 [32-47]	40 [35-50]	30 [27-48]	54 [48-59]	53 [45-59]
Had prior COVID symptoms	51 (26%)	38 (28%)	23 (34%)	6 (23%)	11 (85%)	12 (92%)	18 (22%)	19 (22%)
Median days since dose 2 [IQR]	29 [23-36]	101 [93-107]	28 [22-34]	96 [90-99]	186 [145-211]	134 [109-144]	192 [188-202]	217 [211-230]
Median days since dose 3 [IQR]							-2 [-5-0]	20 [18-22]
Quantifiable NAbT…								
… vs. Alpha								
Yes	199 (100%)	131 (96%)	59 (87%)	24 (92%)	11 (85%)	11 (85%)	70 (88%)	72 (85%)
No	0 (0%)	3 (2.2%)	7 (10%)	1 (3.8%)	0 (0%)	0 (0%)	2 (2.5%)	0 (0%)
(n.d.)	0 (0%)	2 (1.5%)	2 (2.9%)	1 (3.8%)	2 (15%)	2 (15%)	8 (10%)	13 (15%)
… vs. Delta								
Yes	198 (99%)	132 (97%)	52 (76%)	21 (81%)	11 (85%)	11 (85%)	63 (79%)	71 (84%)
No	1 (0.5%)	2 (1.5%)	14 (21%)	4 (15%)	0 (0%)	0 (0%)	9 (11%)	1 (1.2%)
(n.d.)	0 (0%)	2 (1.5%)	2 (2.9%)	1 (3.8%)	2 (15%)	2 (15%)	8 (10%)	13 (15%)
… vs. Omicron								
Yes	166 (83%)	69 (51%)	25 (37%)	5 (19%)	13 (100%)	13 (100%)	34 (42%)	82 (96%)
No	33 (17%)	67 (49%)	43 (63%)	21 (81%)	0 (0%)	0 (0%)	46 (57%)	2 (2.4%)
(n.d.)	0 (0%)	0 (0%)	0 (0%)	0 (0%)	0 (0%)	0 (0%)	0 (0%)	1 (1.2%)
Median NAbT [IQR]								
Alpha	600 [384-1141]	224 [143-427]	168 [58-387]	111 [61-172]	1709 [1061-5120]	1509 [1145-3701]	126 [80-299]	1258 [941-1786]
Delta	301 [171-572]	125 [82-214]	71 [41-180]	51 [41-112]	858 [531-1221]	974 [681-1246]	68 [46-140]	626 [472-1032]
Omicron	122 [46-173]	*n.d.*	*n.d.*	*n.d.*	*575 [195-654]*	*571 [420-732]*	*n.d.*	*332 [193-596]*
Median Fold-change NAbT [IQR]								
Alpha vs. Omicron	6.9 [4.2-11.3]	*n.d.*	*n.d.*	*n.d.*	4.7 [3.1-7.6]	3.1 [2.5-4.1]	*n.d.*	*3.7 [2.6-7.0]*
Delta vs. Omicron	3.1 [1.9-5.4]	*n.d.*	*n.d.*	*n.d.*	2.0 [1.6-2.5]	2.1 [1.5-2.4]	*n.d.*	*1.9 [1.3-3.0]*

Table 1. A third analysis of the Legacy study (University College London Hospital and the Francis Crick Institute). Relates to NAbT data shown in **Figure 1B, 1D,** and **1E**. (See **Table 2** for data relating to **Figure 1C**.)

(Refers to Figure 1C)

	2-6 wks POST- 2 Vaccine Doses			
	BNT162b2		AZD1222	
	NO prior symp.	YES prior symp.	NO prior symp.	YES prior symp.
	n = 148	n = 51	n = 45	n = 23
Site				
Crick	67 (45%)	27 (53%)	45 (100%)	23 (100%)
UCLH	81 (55%)	24 (47%)	0 (0%)	0 (0%)
Sex				
Female	98 (66%)	38 (75%)	30 (67%)	13 (57%)
Male	50 (34%)	13 (25%)	15 (33%)	10 (43%)
Median age (years) [IQR]	43 [33-52]	40 [32-48]	33 [29-42]	36 [32-40]
Had prior COVID symptoms	0 (0%)	51 (100%)	0 (0%)	23 (100%)
Median days since dose 2 [IQR]	30 [23-37]	29 [22-34]	28 [23-34]	28 [22-36]
Median days since dose 3 [IQR]				
Quantifiable NAbT…				
… vs. Alpha				
Yes	148 (100%)	51 (100%)	40 (89%)	19 (83%)
No	0 (0%)	0 (0%)	5 (11%)	2 (8.7%)
(n.d.)	0 (0%)	0 (0%)	0 (0%)	2 (8.7%)
… vs. Delta				
Yes	147 (99%)	51 (100%)	33 (73%)	19 (83%)
No	1 (0.7%)	0 (0%)	12 (27%)	2 (8.7%)
(n.d.)	0 (0%)	0 (0%)	0 (0%)	2 (8.7%)
… vs. Omicron				
Yes	119 (80%)	47 (92%)	8 (18%)	17 (74%)
No	29 (20%)	4 (7.8%)	37 (82%)	6 (26%)
(n.d.)	0 (0%)	0 (0%)	0 (0%)	0 (0%)
Median NAbT [IQR]				
Alpha	535 [347-1018]	804 [545-1854]	121 [50-193]	450 [234-759]
Delta	255 [154-478]	524 [260-816]	47 [10-87]	209 [161-457]
Omicron	92 [42-158]	165 [122-387]	*n.d.*	118 [29-164]
Median Fold-change NAbT [IQR]				
Alpha vs. Omicron	7.1 [4.8-12.4]	5.1 [3.2-9.0]	11.6 [8.1-30.5]	3.8 [2.9-7.5]
Delta vs. Omicron	3.3 [2.1-6.0]	2.3 [1.4-4.3]	*n.d.*	2.0 [1.4-5.0]

Table 2. Stratification of NAbTs 2-6 weeks post second vaccine dose by vaccine type and prior COVID symptoms. Relates to NAbT data shown in **Figure 1C**.

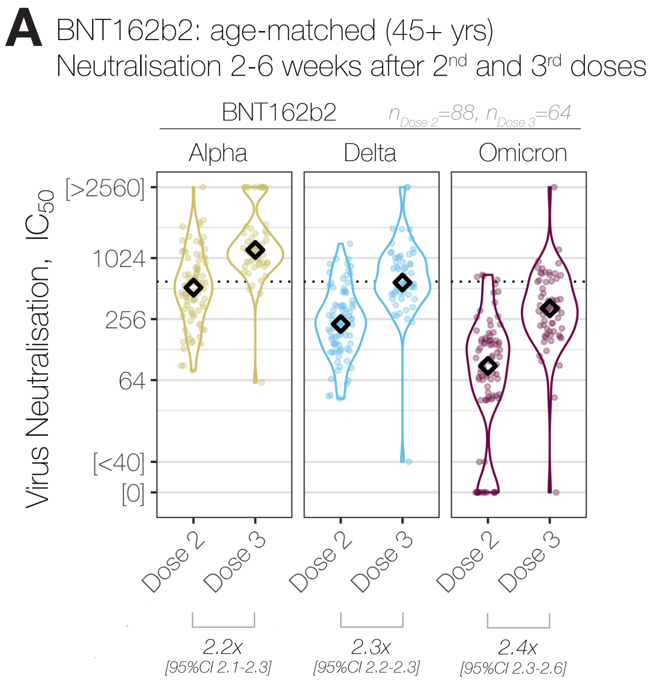

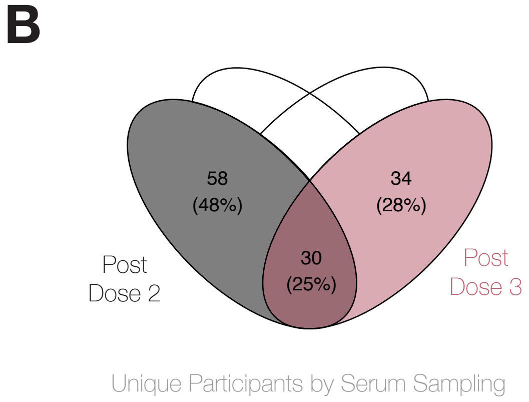

Figure 3. Comparison of NAbTs in an age-matched subset of BNT162b2 vaccine recipients, (**A**) measured 2-6 weeks following second and third dose vaccination. (**B**) Diagram illustrating number of unique participants and overlap between sampling of sera: a total of 152 sera were collected from 122 unique participants aged 45 years and older (see **Table 3**, below).

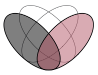

Table 3. Age-matched cohort of BNT162b2 vaccine recipients following second- and third-dose vaccination. Relates to NAbT data shown above in **Figure 3**.

	Age Matched: POST- Vaccine	
	BNT162b2	
	**2 doses**	**3 doses**
	n = 88	n = 64
Site		
Crick	40 (45%)	44 (69%)
UCLH	48 (55%)	20 (31%)
Sex		
Female	56 (64%)	49 (77%)
Male	32 (36%)	15 (23%)
Median age (years) [IQR]	53.0 [48.8-59.0]	55.5 [51.8-61.0]
Had prior COVID symptoms	18 (20%)	14 (22%)
Median days since dose 2 [IQR]	28 [22-37]	214 [211-225]
Median days since dose 3 [IQR]		20 [18-21]
Quantifiable NAbT…		
… vs. Alpha		
Yes	88 (100%)	57 (89%)
No	0 (0%)	0 (0%)
(n.d.)	0 (0%)	7 (11%)
… vs. Delta		
Yes	88 (100%)	56 (88%)
No	0 (0%)	1 (1.6%)
(n.d.)	0 (0%)	7 (11%)
… vs. Omicron		
Yes	71 (81%)	63 (98%)
No	17 (19%)	1 (1.6%)
(n.d.)	0 (0%)	0 (0%)
Median NAbT [IQR]		
Alpha	523 [315-829]	1230 [919-1798]
Delta	229 [145-356]	586 [455-1037]
Omicron	89 [42-158]	327 [193-600]
Median Fold-change NAbT [IQR]		
Alpha vs. Omicron	6.9 [4.3-10.3]	4.1 [2.6-7.1]
Delta vs. Omicron	2.8 [1.9-4.9]	2.0 [1.3-3.1]

Supplementary Methods

Clinical cohort

Two prospective cohorts of Legacy participants were established in January 2021 (NCT04750356). Participants were included if they were an employee of either UCLH or the Francis Crick Institute and had submitted at least one sample for RT-qPCR occupational health testing for COVID-19 using the Crick testing pipeline. Participants consisted of patient-facing healthcare workers at UCLH, who had received at least one dose of a currently licensed COVID-19 vaccine and Crick staff. Participants were sampled at approximately 1 month post-vaccination and invited for follow up visits at approximately 3, 6, and 12 months, and participants were invited for an additional visit approximately 2-4 weeks following a "breakthrough" infection subsequent to receiving two vaccine doses. Additional participants were also recruited following breakthrough infection via publicity coordinated with occupational health testing, and existing participants were requested to report breakthrough infections via study publicity and coordination with occupational health testing; those experiencing breakthrough infections were sent additional nasopharyngeal swabs and RNA analysed to determine the VOC underlying their infection. Following the introduction of third-dose vaccination in September-December 2021, eligible participants were invited to for an additional visit before or at the time of receiving their third dose, and an additional visit approximately 3 weeks afterwards. All participants were sampled at each visit with additional nasopharyngeal RT-qPCR for SARS CoV-2 (in addition to their occupational health testing) to exclude concurrent active infection, blood was collected for live-virus neutralisation assays. Sera from participants were analysed by participants' vaccine dose number, date since vaccine dose, presence of prior COVID symptoms, experience of "breakthrough" infection following second vaccine dose, and age.

Virus variants & culture

The SARS-CoV-2 B.1.1.7 isolate ("Alpha") was hCoV-19/England/204690005/2020, which carries the D614G, Δ69-70, Δ144, N501Y, A570D, P681H, T716I, S982A and D1118H mutations in Spike,[3] and was obtained from Public Health England (PHE), UK, through Prof. Wendy Barclay, Imperial College London, London, UK through the Genotype-to-Phenotype National Virology Consortium (G2P-UK). The B.1.617.2 ("Delta") isolate was MS066352H (GISAID accession number EPI_ISL_1731019), which carries the T19R, K77R, G142D, Δ156-157/R158G, A222V, L452R, T478K, D614G, P681R, D950N mutations in Spike, and was kindly provided by Prof. Wendy Barclay, Imperial College London, London, UK through the Genotype-to-Phenotype National Virology Consortium (G2P-UK). The BA.1 ("Omicron") isolate was M21021166, which carries the A67V, Δ69-70, T95I, Δ142-144, Y145D, Δ211,

L212I, G339D, S371L, S373P, S375F, K417N, N440K, G446S, S477N, T478K, E484A, Q493R, G496S, Q498R, N501Y, Y505H, T547K, D614G, H655Y, N679K, P681H, A701V, N764K, D796Y, N856K, Q954H, N969K, and L981F mutations in Spike, and was kindly provided by Prof. Gavin Screaton, University of Oxford, Oxford, UK through the Genotype-to-Phenotype National Virology Consortium (G2P-UK).

All viral isolates were propagated in Vero V1 cells. Briefly, 50% confluent monolayers of Vero V1 cells were infected with the given SARS CoV-2 strains at an MOI of approx. 0.001. Cells were washed once with DMEM (Sigma; D6429), then 5 ml virus inoculum made up in DMEM was added to each T175 flask and incubated at room temperature for 30 minutes. DMEM + 1% FCS (Biosera; FB-1001/500) was added to each flask. Cells were incubated at 37° C, 5% CO_2 for 4 days until extensive cytopathogenic effect was observed. Supernatant was harvested and clarified by centrifugation at 2000 rpm for 10 minutes in a benchtop centrifuge. Supernatant was aliquoted and frozen at -80°C.

Virus PCR, sequencing, and characterisation

All virus stocks generated for use in neutralisation assays were sequence-validated prior to use. To confirm the identity of the cultured Omicron VOC, Sanger sequencing was used. For Alpha and Delta VOCs, 8ul of viral RNA was prepared for sequencing by the ARTIC method (https://www.protocols.io/view/ncov-2019-sequencing-protocol-v3-locost-bh42j8ye) and sequenced on the ONT GridION platform to >30k reads / sample. The data was demultiplexed and processed using the viralrecon pipeline (https://github.com/nf-core/viralrecon). RNA was extracted from nasopharyngeal swabs taken at time of breakthrough infection (available for 17/26 breakthrough cases) and subjected to RT-qPCR analysis (TaqPath COVID-19 CE-IVD Kit, ThermoFisher) to confirm SARS-CoV-2 infection and assess the presence of S-gene target failure (SGTF). The date of infection (late June – November 2021), SGTF, and the UK variant prevalence (https://www.gov.uk/government/publications/covid-19-variants-genomically-confirmed-case-numbers/variants-distribution-of-case-data-2-july-2021) were used to assign the likely variant (Alpha or Delta) that caused breakthrough infection.

High-throughput live virus microneutralisation assay

High-throughput live virus microneutralisation assays were performed as described previously[5]. Briefly, Vero E6 cells (Institut Pasteur) at 90-100% confluency were infected with given SARS-CoV-2 variants at an MOI of ~1 (variants in 384-well format, in the presence of serial dilutions of patient serum samples or synthetic monoclonal antibodies. After infection, cells were fixed with 4% final Formaldehyde, permeabilised with 0.2% TritonX-100, 3% BSA in PBS (v/v), and stained for SARS-CoV-2 N protein using Alexa488-labelled-CR3009

antibody produced in-house and cellular DNA using DAPI[7]. Whole-well imaging at 5x was carried out using an Opera Phenix (Perkin Elmer) and fluorescent areas and intensity calculated using the Phenix-associated software Harmony (Perkin Elmer). Inhibition was estimated from the measured area of infected cells/total area occupied by all cells. The inhibitory profile of each serum sample was estimated by fitting a 4-parameter dose response curve executed in SciPy. Neutralising antibody titres are reported as the fold-dilution of serum required to inhibit 50% of viral replication (IC_{50}), and are further annotated if they lie above the quantitative (complete inhibition) range, below the quantitative range but still within the qualitative range (i.e. partial inhibition is observed but a dose-response curve cannot be fit because it does not sufficiently span the IC_{50}), or if they show no inhibition at all.

Data analysis, statistics, and availability

Study data were collected and managed using REDCap electronic data capture tools hosted at University College London[8,9]. Data were exported from REDCap into R for visualisation and analysis. Neutralising antibody titres are reported as IC_{50} values. As described above, for each serum sample, four dilutions (1:40, 1:160, 1:640, 1:2560) are assayed in duplicate. All 8 points are used to fit a 4 parameter curve, and the IC_{50} (the fold-dilution corresponding to 50% viral inhibition), is reported. IC_{50} values below 40 and above 2560 are reported as 'weak' or 'complete' inhibition, as described above. For plotting and analysis, winsorizing was used: IC_{50} values above the quantitative limit of detection of the assay (>2560) were recoded as 5120; IC50 values below the quantitative limit of the assay (< 40) but within the qualitative range were recoded as 10; data below the qualitative range (i.e. no response observed) were recoded as 5. These changes do not affect any statistical parameters considered in the quantitative analyses below (i.e. non-parametric approaches, rather than parametric approaches such as geometric means are used, and these winsorized values lie outside the median and IQR).

- Data analysis was carried out using the *dplyr* package in R. Summary descriptions of the clinical cohort and of measured NAbTs were generated using the *gtsummary* package in R and its *tbl_summary* function, specifying calculation of median and IQR for continuous variables using the argument *statistic = all_continuous() ~ "{median} [{p25}-{p75}]"*
- The 95% confidence interval for the median fold-difference of serum viral neutralisation IC_{50} between different variants following two-dose vaccination was determined using bootstrap statistics, implemented in R using the *infer* package using the *generate* function, specifying the *type = "bootstrap"* and *reps = 5000* arguments, and *get_confidence_interval* function, without modification of their default parameters.

Where a fold-change could not be calculated due to missing paired data across variants, samples were excluded from paired analysis (see **Tables 1-3**, “n.d.”).

- The number of sera that did (or did not) have a quantifiable NAbT of Alpha or Delta vs. Omicron was assessed for statistical significance using Pearson's Chi-squared test with Yates' continuity correction, implemented by the *chisq.test* function in the R *stats* package, without modification of its default parameters.
- The same method was used to assess the significance of the number of sera that did (or did not) have a quantifiable NAbT against Omicron between those who did (or did not) report experiencing prior COVID symptoms, in the cohort of AZD1222 recipients sampled 2-6 weeks following their second vaccine dose.
- The difference in NAbTs between sera from BNT162b2 recipients sampled 2-6 weeks following their second vaccine dose who did (or did not) report experiencing prior COVID symptoms, was assessed for statistical significance using the Wilcoxon rank sum test with continuity correction, implemented by the *wilcox.test* function, specifying the *paired = FALSE* argument, in the R *stats* package, without modification of its default parameters.
- The 95% confidence interval for the median fold-difference of serum viral neutralisation IC_{50} between different variants following three-dose vaccination was determined as described above for two-dose vaccination.
- The 95% confidence interval for the fold-increase in the median serum viral neutralisation IC_{50} of two-dose vs. three-dose recipients (unpaired), in older BNT162b2 participants (>= 45 yrs), was determined using bootstrap statistics using the *boot* package in in R, specifying *rep=5000* and *strata* arguments for the *boot* function, and the *type="basic"* and *conf=0.95* arguments for the *boot.ci* function, without further modification of their default parameters.

Graphs for participant neutralisation titres were generated using the *ggplot2* package in R, Venn diagrams illustrating the overlap between serum sampling and unique participants were generated using the *ggvenn* package in R, and graphs for monoclonal antibody neutralisation were generated in Prism (GraphPad). All data (anonymised) and full R code to produce all figures and statistical analysis presented in this manuscript are freely-available online on Github: https://github.com/davidlvb/Crick-UCLH-Legacy-Omicron-2021-12

Ethics

The Legacy study was approved by London Camden and Kings Cross Health Research Authority (HRA) Research and Ethics committee (REC) IRAS number 286469 and sponsored by University College London.

Role of the funding source

This work was undertaken at UCLH/UCL who received a proportion of funding from the National Institute for Health Research (NIHR) University College London Hospitals Department of Health's NIHR Biomedical Research Centre (BRC). EW, VL and BW are supported by the Centre's funding scheme. This work was supported jointly by the BRC and core funding from the Francis Crick Institute, which receives its funding from Cancer Research UK, the UK Medical Research Council, and the Wellcome Trust. DLVB is additionally supported by the Genotype-to-Phenotype National Virology Consortium (G2P-UK) via UK Research and Innovation and the UK Medical Research Council. The funders of the study had no role in study design, data collection, data analysis, data interpretation, or writing of the report. The corresponding authors had full access to all the data and the final responsibility to submit for publication.

Declaration of interests

CSw reports interests unrelated to this Correspondence: grants from BMS, Ono-Pharmaceuticals, Boehringer-Ingelheim, Roche-Ventana, Pfizer and Archer Dx, unrelated to this Correspondence; personal fees from Genentech, Sarah Canon Research Institute, Medicxi, Bicycle Therapeutics, GRAIL, Amgen, AstraZeneca, BMS, Illumina, GlaxoSmithKline, MSD, and Roche-Ventana, unrelated to this Correspondence; and stock options from Apogen Biotech, Epic Biosciences, GRAIL, and Achilles Therapeutics, unrelated to this Correspondence. DLVB reports grants from AstraZeneca unrelated to this Correspondence. All other authors declare no competing interests.

Acknowledgements

This research was funded in whole, or in part, by the Wellcome Trust [FC011104, FC011233, FC001030, FC001159, FC001827, FC001078, FC001099, FC001169]. For the purpose of Open Access, the author has applied a CC BY public copyright licence to any Author Accepted Manuscript version arising from this submission. The authors would like to thank all the study participants, the staff of the NIHR Clinical Research Facility at UCLH including Kirsty Adams and Marivic Ricamara. We would like to thank Jules Marczak, Gita Mistry, Simon Caiden and the staff of the Scientific Technology Platforms (STPs) and COVID-19 testing pipeline at the Francis Crick Institute. We thank Prof. Wendy Barclay of Imperial College and the wider Genotype to Phenotype consortium for the Alpha and Delta strains used in this study, and Max Whiteley and Thushan I de Silva at The University of Sheffield and Sheffield Teaching Hospitals NHS Foundation Trust for providing source material. We thank Prof. Gavin Screaton of the University of Oxford for the Omicron strain used in this study. This work was supported

by the National Institute for Health Research University College London Hospitals Department of Health's NIHR Biomedical Research Centre (BRC), as well as by the UK Research and Innovation and the UK Medical Research Council (MR/W005611/1), and by the Francis Crick Institute which receives its core funding from Cancer Research UK (FC011104, FC011233, FC001030, FC001159, FC001827, FC001078, FC001099, FC001169), the UK Medical Research Council (FC011104, FC011233, FC001030, FC001159, FC001827, FC001078, FC001099, FC001169), and the Wellcome Trust (FC011104, FC011233, FC001030, FC001159, FC001827, FC001078, FC001099, FC001169).

Supplementary References

1. Ng KW, Faulkner N, Cornish GH, et al. Preexisting and de novo humoral immunity to SARS-CoV-2 in humans. *Science* 2020; **370**(6522): 1339-43.

2. Houlihan CF, Vora N, Byrne T, et al. Pandemic peak SARS-CoV-2 infection and seroconversion rates in London frontline health-care workers. *Lancet* 2020; **396**(10246): e6-e7.

3. Brown JC, Goldhill DH, Zhou J, et al. Increased transmission of SARS-CoV-2 lineage B.1.1.7 (VOC 2020212/01) is not accounted for by a replicative advantage in primary airway cells or antibody escape. *bioRxiv* 2021: 2021.02.24.432576.

4. Cele S, Gazy I, Jackson L, et al. Escape of SARS-CoV-2 501Y.V2 from neutralization by convalescent plasma. *Nature* 2021; **593**(7857): 142-6.

5. Faulkner N, Ng KW, Wu M, et al. Reduced antibody cross-reactivity following infection with B.1.1.7 than with parental SARS-CoV-2 strains. *bioRxiv* 2021: 2021.03.01.433314.

6. Rihn SJ, Merits A, Bakshi S, et al. A plasmid DNA-launched SARS-CoV-2 reverse genetics system and coronavirus toolkit for COVID-19 research. *PLoS Biol* 2021; **19**(2): e3001091.

7. van den Brink EN, Ter Meulen J, Cox F, et al. Molecular and biological characterization of human monoclonal antibodies binding to the spike and nucleocapsid proteins of severe acute respiratory syndrome coronavirus. *J Virol* 2005; **79**(3): 1635-44.

8. Harris PA, Taylor R, Minor BL, et al. The REDCap consortium: Building an international community of software platform partners. *J Biomed Inform* 2019; **95**: 103208.

9. Harris PA, Taylor R, Thielke R, Payne J, Gonzalez N, Conde JG. Research electronic data capture (REDCap)--a metadata-driven methodology and workflow process for providing translational research informatics support. *J Biomed Inform* 2009; **42**(2): 377-81.
